# Immunological profiling for short-term predictive analysis in PD-1/PD-L1 therapy for lung cancer

**DOI:** 10.1186/s12885-024-12628-5

**Published:** 2024-07-18

**Authors:** Yun Wang, Rujia Chen, Zhenzhou Guo, Wei Wei, Ting Wang, Renren Ouyang, Xu Yuan, Yutong Xing, Feng Wang, Shiji Wu, Hongyan Hou

**Affiliations:** 1 Department of Laboratory Medicine, Tongji Hospital, Tongji Medical College, Huazhong University of Science and Technology, Jiefang Road 1095, Wuhan, 1095, 430030, 430030 China; 2 Department of Laboratory Medicine, Xinfeng County People’s Hospital, Ganzhou, China

**Keywords:** Lung cancer, PD-1, PD-L1, CD4 + T cells, Treg Cells

## Abstract

**Background:**

Immune checkpoint inhibitors, such as anti-programmed cell death-1 (PD-1) and PD-1 ligand-1 (PD-L1) antibodies, have achieved breakthrough results in improving long-term survival rates in lung cancer. Although high levels of PD-L1 expression and tumor mutational burden have emerged as pivotal biomarkers, not all patients derive lasting benefits, and resistance to immune checkpoint blockade remains a prevalent issue. Comprehending the immunological intricacies of lung cancer is crucial for uncovering the mechanisms that govern responses and resistance to immunomodulatory treatments. This study aimed to explore the potential of peripheral immune markers in predicting treatment efficiency among lung cancer patients undergoing PD-1/PD-L1 checkpoint inhibitors.

**Methods:**

This study enrolled 71 lung cancer patients undergoing PD-1/PD-L1 inhibitor therapy and 20 healthy controls. Immune cell subsets (CD4 + T cells, CD8 + T cells, B cells, NK cells, and NKT cells), phenotypic analysis of T cells and B cells, and PMA/Ionomycin-stimulated lymphocyte function assay were conducted.

**Results:**

Lung cancer patients exhibited significant alterations in immune cell subsets, notably an increased percentage of Treg cells. Post-treatment, there were substantial increases in absolute numbers of CD3 + T cells, CD8 + T cells, and NKT cells, along with heightened HLA-DR expression on CD3 + T and CD8 + T cells. Comparison between complete remission and non-complete remission (NCR) groups showed higher Treg cell percentages and HLA-DR + CD4 + T cells in the NCR group.

**Conclusion:**

The study findings suggest potential predictive roles for immune cell subsets and phenotypes, particularly Treg cells, HLA-DR + CD4 + T cells, and naïve CD4 + T cells, in evaluating short-term PD-1/PD-L1 therapy efficacy for lung cancer patients. These insights offer valuable prospects for personalized treatment strategies and underscore the importance of immune profiling in lung cancer immunotherapy.

**Supplementary Information:**

The online version contains supplementary material available at 10.1186/s12885-024-12628-5.

## Introduction

Lung cancer (LC) is a significant global public health issue, representing the foremost cause of cancer-related fatalities on a worldwide scale. It continues to stand as the primary contributor to global cancer-related deaths, accounting for 18.7% of total cancer mortality, imposing substantial societal and economic burdens [1, 2]. Despite various treatments including surgery, chemotherapy, and radiation, there is a need for more effective methods, especially for advanced stages [3]. Activating the immune system for therapeutic purposes in cancer treatment has long been a goal of immunologists and a select group of oncologists. The introduction of therapies designed to target inhibitory receptors expressed by T lymphocytes has ushered in a transformative era in the field of cancer treatment [4].

Immune cells are essential components of the tumor stroma and play a pivotal role in this process [5]. Immune checkpoint inhibitors (ICI), such as anti-programmed cell death-1 (PD-1)/PD-1 ligand-1 (PD-L1) antibodies, have achieved breakthrough results in long-term survival in many cancers [6–11]. While high levels of PD-L1 expression [12] and tumor mutational burden [13] have become pivotal biomarkers, the varying state of T-cell immunity, which greatly differs from one patient to another, has resulted in notable disparities in the effectiveness of anti-tumor treatments. Their effectiveness varies among patients due to the complex tumor microenvironment (TME), a significant proportion of patients do not benefit from these therapies [14–16].

Thorough immunoprofiling provides a solid foundation for evaluating eligibility for tailored therapies. Emerging approaches, like multiplexed slide-based analysis, offer personalized treatment strategies to enhance immunomodulatory efficacy in lung cancer [17]. Prior investigations have substantiated the link between peripheral blood lymphocytes and the prognosis of lung cancer patients as well as the effectiveness of drug therapy [18, 19]. In this study, we aimed to investigate the connection between peripheral blood lymphocyte subsets, phenotypes, tumor markers, and the effectiveness of combining Lenvatinib with PD-1/PD-L1 inhibitors, providing insights for personalized treatment and immunotherapy strategies.

## Materials and methods

### Patients and sample collection

A total of 71 patients (58 males and 13 females) diagnosed with lung cancer treated with PD-1/PD-L1 checkpoint inhibitors were included in this study from December 2021 to October 2022. None of these patients had previous evidence for an underlying immunodeficiency, and none of them received previous immunosuppressive drugs or other systemic therapy such as chemotherapy and targeted therapy. The patients whose tumors harbored oncogenic alternations (particularly EGFR mutations and ALK and ROS1 rearrangements) are also excluded. Best response to systemic therapies, defined as a complete or partial response achieved at least once during the course of therapy, was assessed locally using RECIST v1.1 criteria [20]. Lung cancer patients were divided into complete response (CR) and non-complete response (NCR) groups which include partial response, stable disease and progressive disease. This classification was based on the effectiveness after two-cycle treatment. Among the 71 LC patients, 45 patients had complete monitoring data available both before treatment and after two-cycle of treatment: 15 were in the CR group, and 30 were in the NCR group. The remaining 26 patients did not have continuous monitoring results, and therefore were not included in the CR and NCR groups. Moreover, 20 healthy controls (HCs, 13 males and 7 females) were included in the study as age- and sex-matched comparison groups determined by interview and physical examination. This study was approved by the ethical committee of Tongji Hospital, Tongji Medical College, Huazhong University of Science and Technology (TJ-IRB20230601).

Lung cancer patients have fresh venous blood collected at three distinct time points: upon admission, after two-cycle treatment and four-cycle treatment, all within 24 h. Healthy control subjects also have fresh venous blood collected. 2 mL venous blood sample is collected in a sodium heparin tube (BD Biosciences, San Jose, USA) for the detection of lymphocyte subsets, phenotypes, and functions, as well as the detection of liver and kidney function levels. 2 mL venous blood sample is collected in a serum separation tube (BD Biosciences, San Jose, USA) for the determination of serum tumor marker levels. These blood samples are stored at room temperature of 20–25℃ and tested within 24 h. 1 mL venous blood sample is collected in EDTA-K2 tube (BD Biosciences, San Jose, USA) for the assessment of routine blood tests, which are conducted within 4 h at room temperature of 20–25℃.

### TBNK lymphocyte counting

The percentages and absolute numbers of CD4 + T cells, CD8 + T cells, B cells, NK cells and NKT cells were assessed using TruCOUNT tubes and BD Multitest 6-color TBNK Reagent Kit (BD Biosciences, San Jose, USA) according to the manufacturer’s instructions. In brief, 50 µL of whole blood was labeled with six-color TBNK Ab cocktail regent for 15 min at room temperature. Subsequently, 450 µL of FACS lysing solution was added, and the samples were analyzed using a FACSCanto flow cytometer equipped with FACSCanto clinical software (BD Biosciences, San Jose, USA).

### Lymphocyte phenotypes

The phenotypes of T cells and B cells were analyzed by flow cytometry. Three panels were designed and the following monoclonal antibodies (BD Biosciences, San Jose, USA) and reagents were added to 100 µL whole blood. The antibodies in panel 1 were anti-CD45-PerCP, anti-CD3-APC-H7, anti-CD4-V450, anti-CD8-PE/Cy7, anti-CD28-PE, and anti-HLA-DR-APC. The antibodies in panel 2 were anti-CD45-PerCP, anti-CD3-APC-H7, anti-CD4-V500C, anti-CD45RA-FITC, anti-CD8-PE/Cy7, anti-CCR7-PE, anti-CD25-APC, and anti-CD127-BV421. The subsets of B cells analyzed by panel 3 were detected using anti-CD38-FITC, anti-CD19-PE/Cy7, anti-CD27-PerCP and CD45-V500C, and anti-IgD-APC. Isotype controls with irrelevant specificities were included as negative controls. All of these cell suspensions were incubated for 20 min at room temperature. After lysing red blood cells with lysing solution, the pellets were washed, re-suspended in 300 µL PBS, and subsequently analyzed using a FACS Canto flow cytometer.

### PMA/Ionomycin-stimulated lymphocyte function assay

The procedures are described in brief as following: (1) 100 µL of whole blood was diluted with 400 µL of IMDM medium (Gibco, (Life Technologies Corporation, NY, USA )) in 12 × 75 mm polystyrene round-bottom tubes with caps ( BD Biosciences, San Jose, USA); (2) the diluted whole blood was incubated in the presence of Leukocyte Activation Cocktail (Becton Dickinson GolgiPlug™, (BD Biosciences, San Jose, USA)) for 4 h at 37℃ with 5% CO2; (3) after stimulation, 300 µL of supernatant was aspirated, the cells were labeled with antibodies (anti-CD45, anti-CD3, anti-CD4, anti-CD56, and anti-CD8) and incubated for 15 min at room temperature; (4) after lysis of erythrocytes, the cell suspensions were fixed and permeabilized with Fixation/Permeabilization Buffer for 20 min at room temperature; (5) after washing, the cells were stained with intracellular anti-IFN-γ antibody for 20 min at room temperature; (6) the cells pellets were resuspended in 200 µL PBS and analyzed with FACSCanto flow cytometer. The percentages of IFN-γ + cells in different cell subsets were defined as the function of them (e.g., the percentage of IFN-γ + cells in CD3 + CD4 + CD8- cells was regarded as the function of CD4 + T cells; the percentage of IFN-γ + cells in CD3 + CD4-CD8 + cells was regarded as the function of CD8 + T cells; the percentage of IFN-γ + cells in CD3-CD56 + cells was regarded as the function of NK cells).

### Tumor marker and routine blood levels analysis

We determined the levels of squamous cell carcinoma antigen (SCC) and carcinoembryonic antigen (CEA) using the electrochemiluminescence method with Alinity instrumentation (Abbott, Chicago, USA). Similarly, the concentrations of neuron-specific enolase (NSE) and cytokeratin-19 fragment (Cyfra 21 − 1) were quantified with the same electrochemiluminescence technique, employing Roche Diagnostics (Roche Diagnostic, Basel, Switzerland). These assays were performed according to the manufacturer’s instructions to ensure accurate and reliable measurements of tumor marker levels in the serum samples. Routine blood levels were detected by the Sysmex XN-9000 (Sysmex, Kobe, Japan). Liver function and kidney function levels were measured using the Roche Cobas c701 Automatic Electrochemiluminescence Immunoassay System (Roche Diagnostic, Basel, Switzerland).

### Statistical analysis

Continuous variables were presented as either mean ± standard deviation (SD) or median (interquartile range (IQR)), depending on the data distribution. The Mann-Whitney U test or one-way ANOVA test was used to compare the variables between groups, as appropriate. Categorical variables were compared using the Chi-square test or Fisher’s exact test, depending on the sample size and expected cell frequencies. These tests were employed to analyze the associations between categorical variables in the study. Receiver operating characteristic (ROC) curve analysis was performed to identify the optimal cutoff values for parameters, aiming to achieve the highest sensitivity and specificity. The most important lymphocyte subpopulations for predicting CR were screened using stepwise multivariate logistic regression analysis. The decision curve analyses (DCA) were utilized to evaluate the net benefit of the logistic regression. Unsupervised hierarchical cluster analysis was conducted to identify clusters of patients exhibiting similar immune patterns. This analysis was performed using the R package “pheatmap” and the results were represented as a dendrogram. Statistical analyses were performed using GraphPad Prism version 9.5 (San Diego, CA, USA), SPSS version 22.0 (Chicago, IL, USA) and Beckman Coulter DxAI platform (www.xsmartanalysis.com/beckman/login/, (Beckman Coulter, CA, USA)). Statistical significance was determined as *p* < 0.05.

## Results

### Participant characteristics

This study recruited 71 patients with LC (58 males and 13 females), with the median age of 62 (25th -75th, 56–67) years. Additionally, 20 healthy controls (13 males and 7 females) were included, with a median age of 60 years (25th -75th, 54–66). The main clinical and biological characteristics of the participants were summarized in Table 1. The majority of the patients had stage IV disease (71.8%), with only one patient at stage I (1.4%). Among the 71 LC patients, 15 were in the CR group, and 30 were in the NCR group. The remaining patients did not have continuous monitoring results. In the baseline analysis of patients with SCLC and NSCLC, there were no significant differences in the results of lymphocyte cell subsets, phenotypes and function. Among the tumor marker results, except for a significant difference in NSE (*p* = 0.047), there were no statistical differences in CEA, Cyfra 21 − 1 and SCC (Supplementary Table 1). Additionally, no significant differences were observed in tumor markers, lymphocyte subsets, cell phenotypes, and their functions when comparing treatments with PD-1 and PD-L1 inhibitors (Supplementary Table 2 and Supplementary Table 3). These results indicate that both PD-1 and PD-L1 drugs affect peripheral immune populations similarly within our study population.

**Table 1:** Clinical characteristics

Parameters	**LC (** *n* ** = 71)**
Age, median (IQR), year	62 (56–67)
Sex	
Male, *n* (%)	58 (81.7%)
Female, *n* (%)	13 (18.3%)
Pathological stage	
I	1 (1.4%)
II	4 (5.6%)
III	15 (21.2%)
IV	51 (71.8%)
Classification	
NSCLC	56 (78.9%)
SCLC	15 (21.1%)
Immunotherapy	
PD-1	52 (73.2%)
PD-L1	19 (26.8%)

LC, lung cancer; NSCLC, non–small-cell lung cancer; SCLC, small-cell lung cancer; PD-1, Programmed cell death-1; PD-L1, Programmed death-ligand 1

### The baseline immune characteristics of patients with LC

**Fig. 1:**
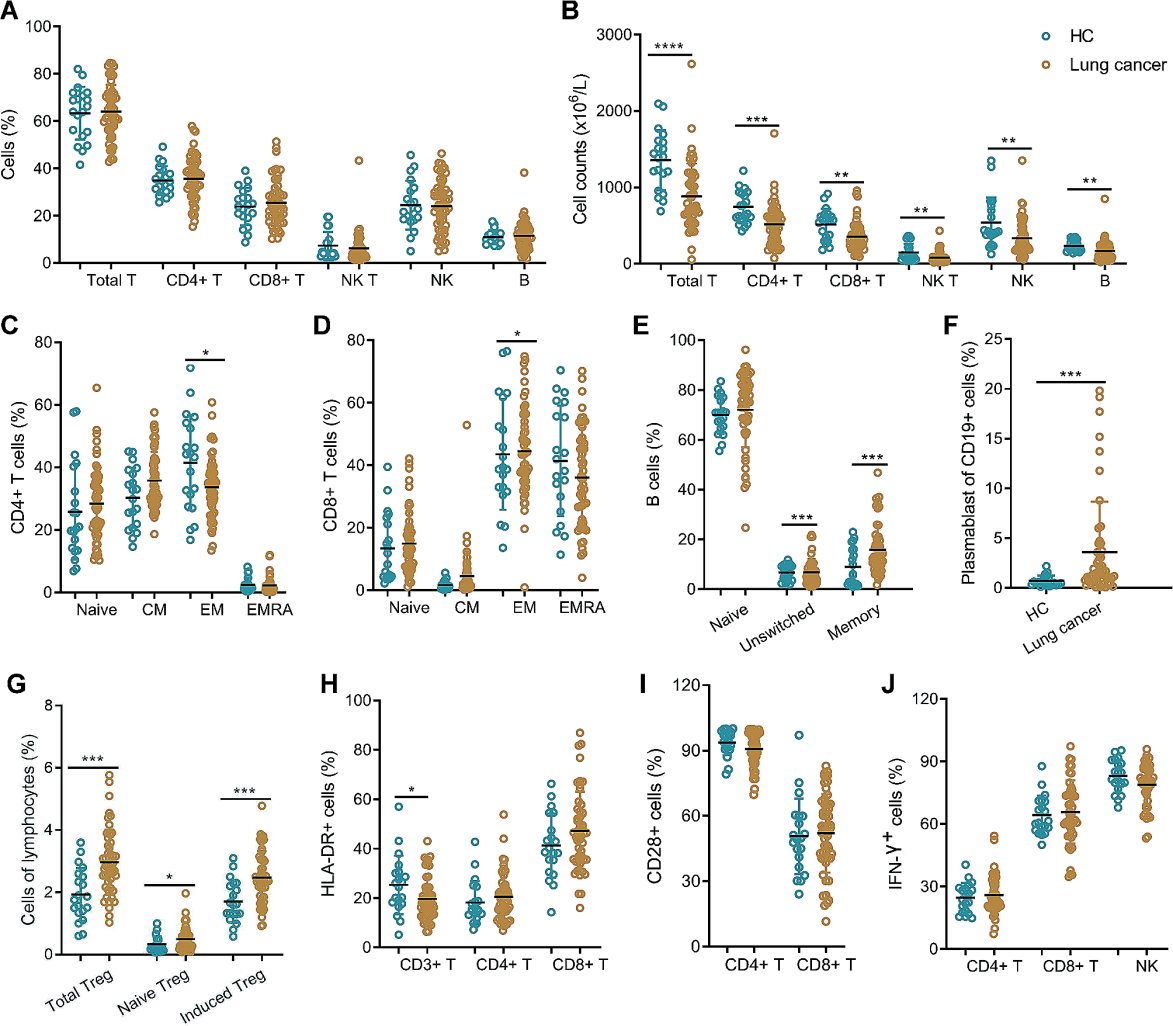
The lymphocyte subsets and immunophenotype characteristics. Circulating lymphocytes in patients diagnosed with lung cancer (LC) and healthy controls (HCs) were analyzed using flow cytometer. **(A, B)** The percentages and absolute numbers of T cells, B cells, NK cells and NKT cells in different groups were expressed as mean with standard deviation (SD). **(C, D)** CD4 + and CD8 + Naïve (Naïve, CCR7 + CD45RA+), central memory (CM, CCR7 + CD45RA−), effector memory (EM, CCR7 − CD45RA−), and terminally differentiated effector memory (EMRA, CCR7 − CD45RA+) T cell subsets were shown. The percentages of CD4 + T and CD8 + T cell subtypes in different groups were expressed as mean with SD. **(E, F)** The percentages of B cell naïve (IgD + CD27−), unswitched (IgD + CD27+), memory (IgD − CD27+), and plasmablast cells (CD27 + CD38high) from LC patients and HCs were expressed as mean with SD. **(G-I)** The percentages of Treg cells, HLA-DR and CD28 positive cells in CD4 + and CD8 + T cells from LC patients and HCs were expressed as mean with SD. **(J)** PMA/Ionomycin-stimulated lymphocyte function. LC, lung cancer; HCs, healthy controls; Blue circle points represent HCs, and orange circle points represent LC patients. *p < 0.05, **p < 0.01, ***p < 0.001, ****p < 0.0001

Patients with LC exhibited dysregulation of immune cell populations compared to HCs. While there were no significant differences in the percentages of immune cell subsets, analysis of lymphocyte subset absolute numbers revealed significant impairments (Fig. 1A-B). To identify the naïve/memory phenotypes of T cells, the expression of CCR7 and CD45RA was analyzed. The results demonstrated a decreased percentage of effector memory (EM) subtype (CCR7-CD45RA-) on CD4 + T cells and an increased percentage of the EM subtype on CD8 + T cells in LC patients (Fig. 1C-D). Notably, LC patients exhibited significant alterations in B cell subpopulations. The percentages of memory (CD27 + IgD-) B cells and plasmablast (CD27 + CD38+) cells were markedly increased in LC patients compared to HCs (Fig. 1E-F). Additionally, the percentages of regulatory T (Treg) cells in LC patients were significantly increased compared to HCs, with both naïve and induced Treg showing increased numbers (Fig. 1G). The expressions of CD28 and HLA-DR on CD4 + T cells and CD8 + T cells showed no significant difference between LC patients and HCs, but the expression of HLA-DR on CD3 + T cells was found to be decreased in LC patients (Fig. 1H-I). Furthermore, we also assessed immune function in LC patients, but no significant difference was found between LCs and HCs (Fig. 1J).

### Alterations in lymphocyte subsets, phenotypes and tumor marker levels during treatment in LC patients

**Fig. 2:**
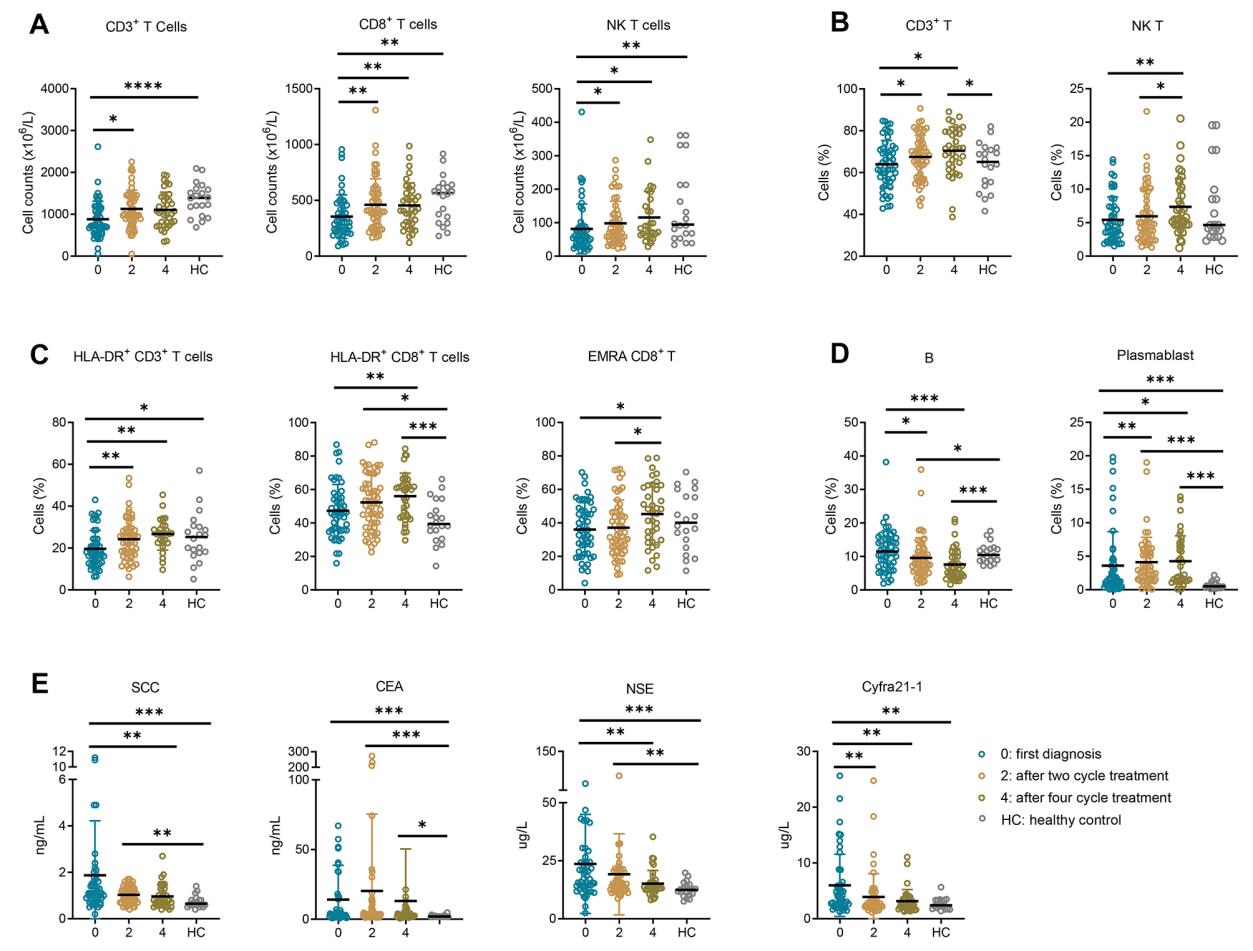
Changes in lymphocyte subsets, phenotypes and tumor markers during treatment in lung cancer patients and healthy control. **(A)** The absolute counts of CD3 + T cells, CD8 + T cells and NKT cells at the first diagnosis, after two-cycle treatment and after four-cycle treatment. **(B, C)** The percentages of CD3 + T cells, NKT cells, HLA-DR + CD3 + T cells, HLA-DR + CD8 + T cells and terminally differentiated effector memory (EMRA, CCR7 − CD45RA+) CD8 + T cells at the first diagnosis, after two-cycle treatment and after four-cycle treatment. **(D)** The percentages of B cells and plasmablast cells at the first diagnosis, after two-cycle treatment and after-four cycle treatment. **(E)** The tumor markers (SCC, CEA, NSE and Cyfra 21 − 1) at the first diagnosis, after two-cycle treatment and after four-cycle treatment. Data were expressed as mean with SD; CEA, carcino-embryonic antigen; NSE, neuro-specific enolase; Cyfra 21 − 1, cytokeratin 19; SCC, squamous cell carcinoma antigen; Blue circle points represent at the first diagnosis, orange circle points represent after two-cycle treatment and brown circle points represent after four-cycle treatment, gray circle points represent healthy control. *p < 0.05, **p < 0.01, ***p < 0.001, ****p < 0.0001

After treatment, there were significant alterations in the cell subsets and phenotypes. The absolute numbers of CD3 + T cells, CD8 + T cells and NKT cells, as well as the percentage of CD3 + T cells, were all significantly increased after two-cycle treatment. However, no significant differences were observed between the effects of two-cycle and four-cycle treatment. The percentage of NKT cells exhibited an increase after four-cycle treatment (Fig. 2A-B). After four-cycle treatment, the terminally differentiated effector memory (EMRA) CD8 + T cells and the expressions of HLA-DR + on CD3 + T cells and CD8 + T cells increased significantly (Fig. 2C). As for B cells, the percentages of B cells decreased significantly and the percentage of plasmablast increased (Fig. 2D). The tumor markers levels were also observed during treatment. Cyfra 21 − 1 exhibited the earliest response, showing a significant reduction after two-cycle treatment, whereas SCC and NSE showed significant reductions after four-cycle treatment (Fig. 2E).

### Changes in lymphocyte subsets and phenotypes during treatment between the CR and NCR groups

**Fig. 3:**
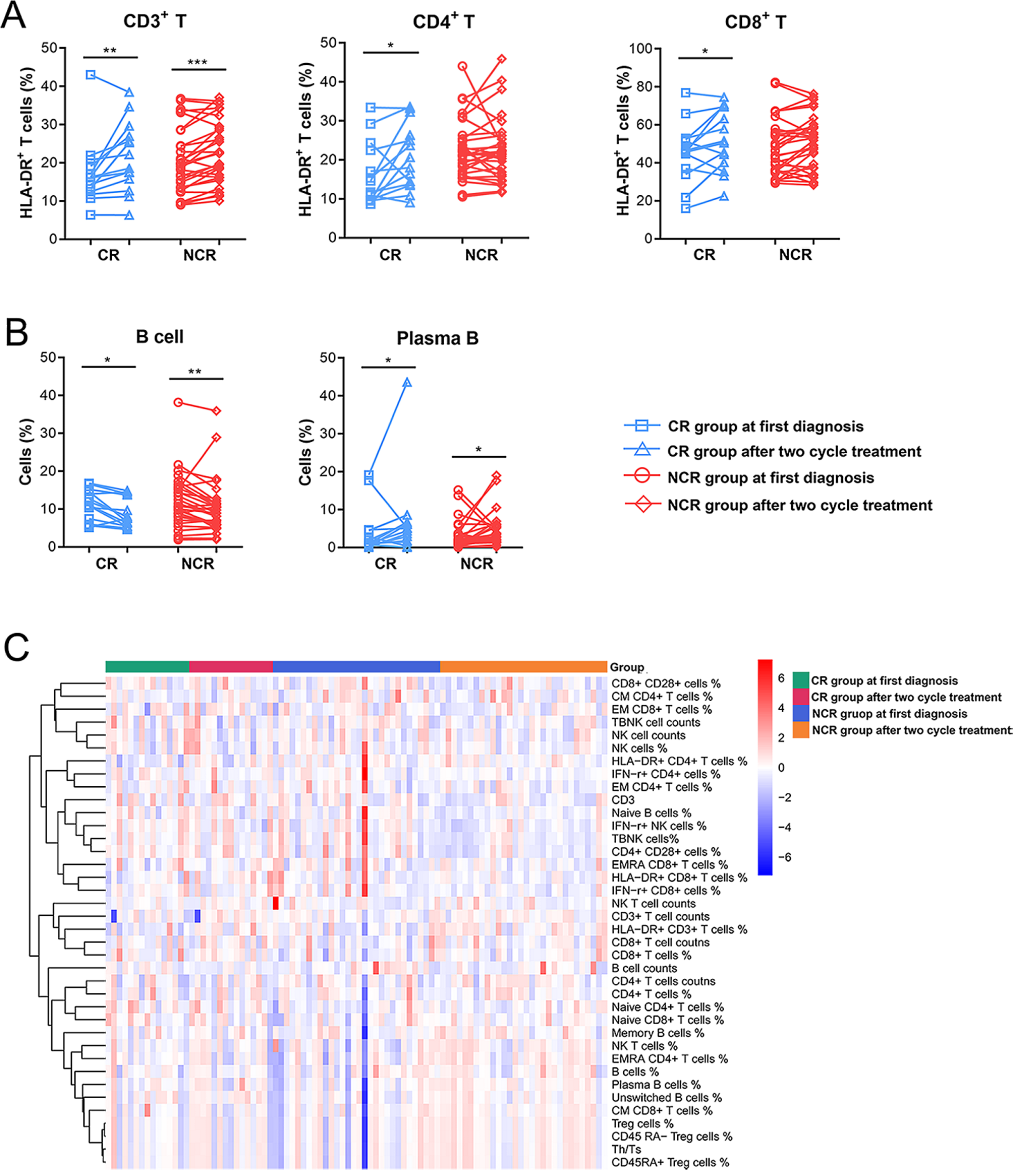
Changes in lymphocyte subsets and phenotypes during treatment between the CR and NCR groups. **(A)** The percentages of HLA-DR + CD3 + T cells, HLA-DR + CD4 + T cells and HLA-DR + CD8 + T cells changes from first diagnosis to after two-cycle treatment. **(B)** The percentages of B cells and plasmablast cells changes from first diagnosis to after two-cycle treatment. **(C)** Hierarchical cluster analysis of immune indicators in CR and NCR groups at first diagnosis and after two cycle treatment. CR, complete remission; NCR, non-complete remission. *p < 0.05, **p < 0.01, ***p < 0.001

We aimed to determine whether variations in lymphocyte subsets and phenotypes could be associated with therapeutic effects during the therapy. A total of 15 CR and 30 NCR patients were enrolled for paired non-parametric Wilcoxon tests. The baseline characteristics and counts of lymphocyte subpopulations between CR and NCR groups were showed in Table 2 and Supplementary Table 4. The percentage of Treg cells and CD45 RA- Treg cells in NCR group were increased significantly, and the expression levels of HLA-DR + on CD4 + T cells were also markedly increased. Blood routine levels and liver function levels showed no significant difference between CR and NCR groups. Interestingly, several positive results were observed between CR and NCR group (Fig. 3). After two-cycle treatment, the expression levels of HLA-DR + on CD3 + T cells, CD4 + T cells and CD8 + T cells and the percentage of plasmablast cells were increase significantly in the CR group. However, the percentage of B cell were decreased. In the NCR group, there was no significant change in the expression levels of HLA-DR + on CD4 + T cells and CD8 + T cells, but the expression of HLA-DR + on CD3 + T cells and the percentage of plasmablast cells were both increased with the percentage of B cells decreased (Fig. 3A-B). A hierarchically clustered heatmap was developed for sample visualization, indicating the impairment of adaptive immune response between CR and NCR group (Fig. 3C). The data of HC, NCR, CR for all significant variables, and a few non-significant ones were showed in Supplementary Table 4.

**Table 2:** Comparison of baseline characteristics between complete remission (CR) and non-complete remission (NCR) groups

Variables	CR (*n* = 15)	NCR (*n* = 30)	*p* value
Age (years)	60.00 (56.00,63.00)	61.00 (56.00,68.00)	0.484
Gender (*n*, %)			
Female	2 (13.33%)	3 (10.00%)	0.737
Male	13 (86.67%)	27 (90.00%)	
Pathological stage			
II	1 (6.67%)	2 (6.67%)	0.727
III	4 (26.67%)	5 (16.67%)	
IV	10 (66.67%)	23 (76.67%)	
Classification			
NSCLC	12 (80.00%)	23 (76.67%)	0.800
SCLC	3 (20.00%)	7 (23.33%)	
Indicator			
Treg cells (%)	2.49 ± 0.67	3.26 ± 1.12	**0.022**
CD45RA- Treg cells (%)	2.07 ± 0.62	2.76 ± 0.86	**0.009**
HLADR + CD4 + T cells (%)	11.69 (9.76,17.07)	21.29 (17.05,25.37)	**0.005**
Naïve CD4 + T cells (%)	32.51 ± 11.18	25.92 ± 9.99	0.056
Albumin (g/L)	41.600 (35.600,44.200)	40.800 (35.500,43.600)	0.813
Lymphocytes (x10^9^/L)	1.090 (0.970,1.530)	1.430 (1.160,1.800)	0.086
Lymphocytes %	16.562 ± 5.953	20.150 ± 8.311	0.177
Monocyte (x10^9^/L)	0.630 (0.430,0.750)	0.540 (0.280,0.790)	0.525
Monocyte %	7.200 (4.900,9.400)	7.000 (5.900,8.100)	0.315
Neutrophil (x10^9^/L)	5.160 (3.990,7.820)	4.730 (3.760,7.720)	0.895
Neutrophil %	73.531 ± 7.212	70.790 ± 10.013	0.389
Platelet (x10^9^/L)	287.000 (281.000,376.000)	235.000 (188.000,281.000)	0.053
RBC (x10^12^/L)	4.299 ± 0.344	4.383 ± 0.698	0.609
Hemoglobin (g/L)	125.231 ± 18.970	132.067 ± 23.976	0.379
WBC (x10^9^/L)	6.510 (5.550,9.600)	7.160 (5.960,11.910)	0.653
LDH (U/L)	212.000 (187.000,235.000)	230.000 (204.000,292.000)	0.140
Total protein (g/L)	73.758 ± 6.097	72.657 ± 5.597	0.587
Amylopsin (U/L)	31.300 (29.300,39.200)	32.000 (29.200,37.000)	0.540
NLR	4.936 (3.560,6.243)	3.238 (2.447,6.229)	0.195
NPR	0.018 (0.014,0.028)	0.020 (0.017,0.034)	0.177

Data are presented as number (%), X ± SD, or median (25th − 75th percentile); NSCLC, non–small-cell lung cancer; SCLC, small-cell lung cancer; RBC, red blood cells; LDH, lactate dehydrogenase; NLR, Neutrophil-to-Lymphocyte Ratio; NPR, Neutrophil-to-Platelet Ratio

### The combined values of HLA-DR + CD4 + T cells , naive CD4 + T cells and Treg cells to predict CR

**Fig. 4:**
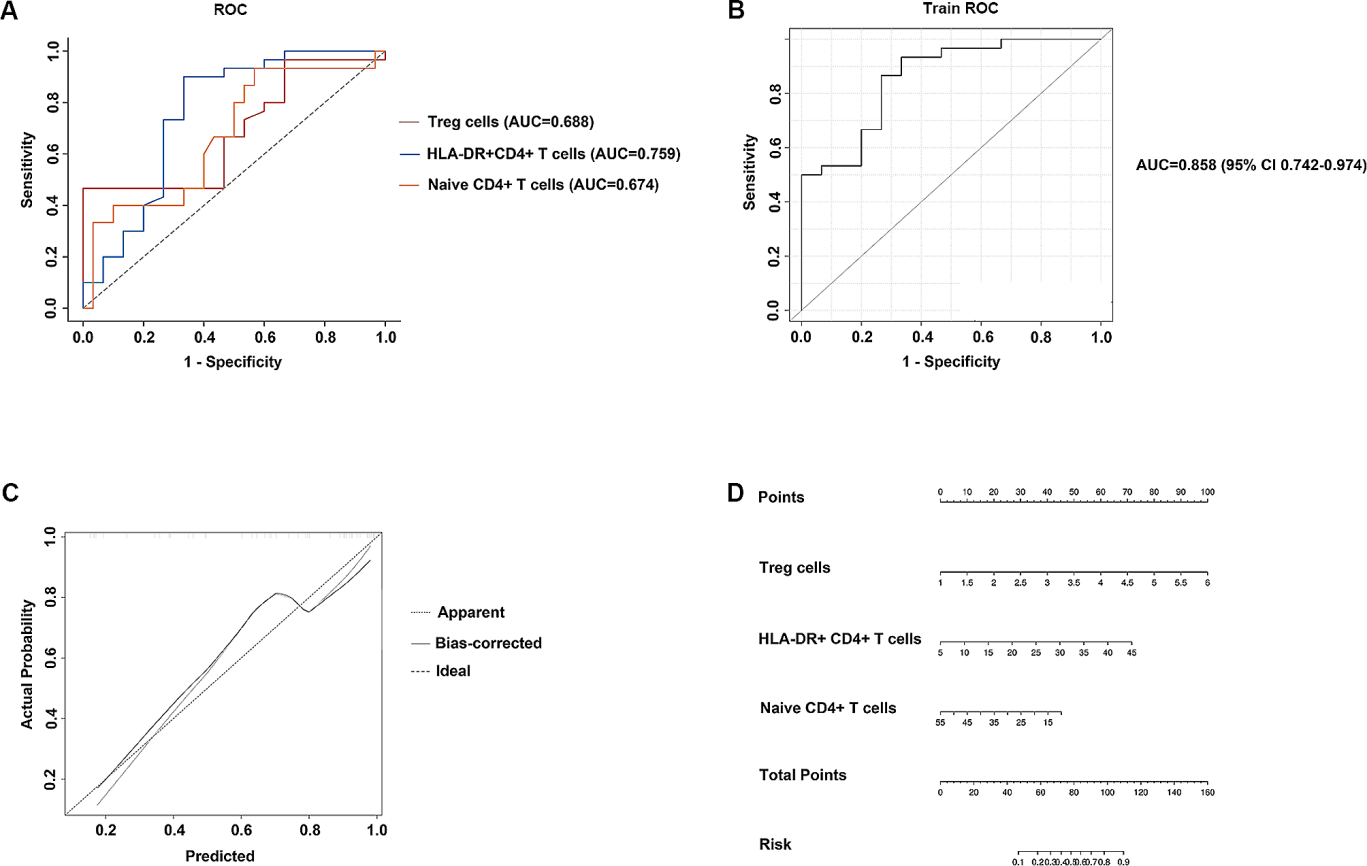
Logistic regression results of combined HLA-DR + CD4 + T cells, naive CD4 + T cells and Treg cells. **(A)** HLA-DR + CD4 + T cells, navie CD4 + T cells and Treg cells independently predicted ROC outcomes for CR and NCR. **(B)** The combined effects of HLA-DR + CD4 + T cells, naive CD4 + T cells, and Treg cells predicted ROC outcomes for CR and NCR. **(C)** The decision curve analysis DCA results of logistic regression. **(D)** Weight scores in the logistic regression model for HLA-DR + CD4 + T cells, naive CD4 + T cells, and Treg cells. ROC, receiver operator characteristic; AUC, area under the curve; CR, complete remission; NCR, non-complete remission

The area under the ROC curve (AUC) was plotted to evaluate the baseline CD4 + T cell values for predicting CR, as shown in Fig. 4. The AUC of naïve CD4 + T cells, HLA-DR + CD4 + T cells and Treg cells were 0.674, 0.759 and 0.688, respectively (Supplementary Table 5). The predictive accuracy of these cut-off values was then evaluated. It seems that the individual prediction ability of each index to CR is not good. Therefore, we combine the three indicators to perform logistic regression to predict CR, and the AUC of the combined detection was 0.858. Naïve CD4 + T cells showed no significant association with odds ratios of 0.932. HLA-DR + CD4 + T cells and Treg cells exhibited a significant association with odds ratio of 1.133 and 4.034, respectively (Table 3). These findings provide insights into the relationships between predictor variables and outcomes.

**Table 3:** Results of logistic regression

Predictor	SE	*p*	Odds Ratio	95% CI
Naive CD4 + T cells %	0.043	0.101	0.932	0.849–1.008
HLA-DR + CD4 + T cells %	0.062	0.043	1.133	1.019–1.306
Treg cells %	0.578	0.016	4.034	1.568–16.740

SE, standard error; CI, confdence interval

## Discussion

Recent improvements in lung cancer treatment precision have not completely resolved the challenges posed by this disease. While PD-1 inhibitors offer hope for advanced lung cancer patients, their effectiveness is limited to less than 20% of cases [21]. Identifying predictive biomarkers for treatment benefit is a critical and urgent need. The aim of this retrospective study was to assess if lymphocyte subsets and phenotypes have the potential to predict the efficacy of PD-1 or PD-L1 treatments in patients with LC.

Our study revealed significant impairments in the absolute numbers of lymphocyte subsets among patients with LC, including a notable increase in the percentage of Treg cells compared to HCs. This suggests a disturbance in the immune environment of lung cancer patients. T cells is an important lymphocyte in anti-tumor immune responses, could generally be divided into several subpopulations. The Treg cell is a subgroup of CD4 + T cells. Some studies have proved that compared with healthy subjects, the percentage of Treg cells in the tumor tissue, peripheral blood, and malignant pleural effusion is higher in patients with lung cancer [22, 23], which were consistent with our results.

With the progress of treatment, the immune status of lung cancer patients has undergone corresponding changes. The absolute numbers of CD3 + T cells, CD8 + T cells and NKT cells were all increased significantly after two cycle treatment. The expression of HLA-DR on CD3 + T cells and CD8 + T cells, and the percentage of EMRA CD8 + T cells were also increased. This typically signifies an enhancement or strengthening of the body’s immune status. It indicates the restoration of the body’s immune system following PD-1/PD-L1 treatment, leading to the reconstruction of anti-tumor immunity [24]. The previous studies indicated that, in lung cancer [25] and renal cell carcinoma [26] patients receiving ICI therapy, CD8 + T cells with effector-like phenotypes (HLA-DR+, CD38+) proliferated, while HLA-DR + CD3 + T cells increase after ICI therapy across various types of tumors [27], aligning with our results. Tumor biomarkers, including CEA, SCC, Cyfra 21 − 1, and NSE, serve as crucial indicators in predicting treatment response in lung cancer patients undergoing ICI therapy [28–30]. Elevated levels have been associated with advanced disease and poor prognosis. Conversely, a decrease in these biomarkers post-treatment correlates with better therapeutic outcomes [31]. Our results show that after treatment, the tumor marker levels were decreased, which also implies either a positive response to treatment or a reduction in the tumor burden.

In comparing the results of CR and NCR, we also found that patients in the NCR group had higher baseline percentages of Treg cells and HLA-DR + CD4 + T cells. At the same time, the percentages of HLA-DR + CD4 + T cells and HLA-DR + CD8 + T cells were increased from baseline after two cycle treatment in the CR group but not altered in the NCR group. The good value of the AUC and DCA performance suggests that Treg cells combined with HLA-DR + CD4 + T cells and Naive CD4 + T cells can be used to predict CR in patients with LC who receive PD-1/PD-L1 inhibitor therapy. Tregs play a pivotal role in the advancement and spread of lung cancer, rendering them significant indicators of less favorable treatment outcomes in individuals with this ailment. Several previous studies have confirmed the accrual or activation of Tregs in lung cancer patients. During the process of carcinogenesis, Tregs are responsible for upholding immune tolerance to self-antigens, facilitating tumor immune evasion, and consequently hindering the anti-tumor immune response [22, 23, 32]. Cancer immunotherapy research has traditionally concentrated on CD8 + T cells within the TME [33, 34]. Nevertheless, reports indicate that the initiation of antitumor immunity is dependent on the presence of MHC class II binding neoantigens, recognized by CD4 + T cells [35]. CD4 + T cells are believed to be the primary catalyst in the cancer immunity cycle, facilitating a continuous provision of CTLs to the TME [35, 36]. HLA-DR acts as a late-stage activation marker for T cells. Its presence in cancer, particularly HLA-DR + T cells in peripheral blood, has yielded contrasting findings. Elevated pretreatment levels of CD8 + HLA-DR + in breast cancer have been correlated with a positive response to neoadjuvant chemotherapy [37]. However, in squamous cell carcinoma of the lung [38] and head and neck cancer [39], increased levels of circulating activated T lymphocytes have been associated with diminished overall survival. Our study showed that low levels of HLA-DR + CD4 + T cells and Treg cells, and high levels of naive CD4 + T cells can predict a positive outcome (CR) for lung cancer patients after treatment. Interestingly, although not statistically significant, patients in the NCR group showed higher baseline levels of HLA-DR + CD8 + T cells. This finding may help explain the contradictory results observed in predicting the prognosis of tumor ICI treatment based on baseline HLA-DR + CD3 + T cells, where baseline HLA-DR + CD4 + T cells predict a positive outcome, while HLA-DR + CD8 + T cells indicate the opposite. However, further research is needed to confirm these observations.

Neutrophil-to-lymphocyte ratio (NLR), neutrophil-to-platelet Ratio (NPR) and lactate dehydrogenase (LDH) have been reported as predictors of prognosis and immune-related adverse events in advanced non-small cell lung cancer treated with PD-1 inhibitors [40, 41]. However, our data indicated that LDH, NLR, and NPR exhibited no significant differences between the CR and NCR groups, suggesting that traditional indicators may not be effective for short-term forecasting. This observation could be attributed to variations in patient populations and the relatively brief monitoring period in our study, in contrast to previous studies that primarily concentrated on long-term predictive performance. Our study places a particular emphasis on short-term predictions, providing valuable insights for clinicians to promptly adjust treatment plans. Our study has several limitations that warrant consideration. Firstly, relatively small sample size, the group of patients with response data available was small, may affect the generalizability of the results. Secondly, the inclusion of stage I and II patients may also affect the applicability of the results. Thirdly, The potential single-center bias may limit the breadth and depth of our conclusions. Future research with larger and multicenter cohorts is needed for a more comprehensive understanding of immune responses in lung cancer patients undergoing therapy.

## Conclusion

This study investigated the immune markers among lung cancer patients treated with PD-1/PD-L1 checkpoint inhibitors revealed promising prospects for predicting treatment response. The analysis of lymphocyte subset absolute numbers in LC patients revealed significant reductions, indicating significant impairments. The alterations observed in lymphocyte subsets and phenotypes, particularly the notable increase in Treg cells and HLA-DR + CD4 + T cells, showcased potential associations with treatment outcomes. Further studies validating these associations could potentially facilitate tailored and more effective treatment strategies for this patient population.

### Electronic supplementary material

Below is the link to the electronic supplementary material.

Supplementary Material 1

Supplementary Material 2

Supplementary Material 3

Supplementary Material 4

Supplementary Material 5

## Data Availability

All data generated or analyzed during this study are included in this published article.
