# Supplementary material for: Immunological profiling for short-term predictive analysis in PD-1/PD-L1 therapy for lung cancer
Source: BMC Cancer. 2024 Jul 18;24:851. doi: 10.1186/s12885-024-12628-5 (PMC11256628; doi:10.1186/s12885-024-12628-5)
Supplement: Supplementary file 1 — Supplementary Material 1 [file 12885_2024_12628_MOESM1_ESM.docx]

| Supplementary Table 1: The initial diagnostic baseline results of SCLC and NSCLC, along with the baseline results of different treatment modalities. | | | | | | |
| --- | --- | --- | --- | --- | --- | --- |
| Parameters | NSCLC (n=41) | SCLC (n=10) | *p* value | PD-1 (n=37) | PD-L1 (n=14) | *p* value |
| Age (years) | 60.000 (56.000,68.000) | 59.000 (54.000,66.000) | 0.552 | 60.000 (56.000,67.000) | 60.000 (56.000,66.000) | 0.849 |
| Gender (n, %) |  |  |  |  |  |  |
| Female | 6(14.634) | 1(10.000) | 0.703 | 5(13.514) | 2(14.286) | 0.943 |
| Male | 35(85.366) | 9(90.000) |  | 32(86.486) | 12(85.714) |  |
| CEA (ng/mL) | 45.652±153.791 | 18.516±39.513 | 0.590 | 48.970±159.444 | 15.335±35.153 | 0.464 |
| NSE (ug/L) | 18.210±8.966 | 63.951±59.436 | 0.047 | 18.476±9.173 | 52.638±56.118 | 0.057 |
| Cyfra21-1 (ug/L) | 7.587±9.246 | 4.306±3.494 | 0.311 | 7.570±9.537 | 5.174±4.105 | 0.413 |
| SCC (ng/mL) | 2.125±2.532 | 0.840±0.284 | 0.123 | 2.092±2.581 | 1.231±1.142 | 0.260 |
| CD3+ T cell counts ( cells/μL) | 760.000 (623.000,1151.000) | 672.000 (641.000,1019.000) | 0.569 | 790.000 (645.000,1151.000) | 672.000 (623.000,1019.000) | 0.387 |
| B cell counts ( cells/μL) | 140.000 (69.000,244.000) | 144.000 (84.000,237.000) | 0.972 | 140.000 (78.000,251.000) | 144.000 (68.000,207.000) | 0.612 |
| CD4+ T cell+ counts ( cells/μL) | 514.000 (311.000,648.000) | 441.000 (372.000,640.000) | 0.713 | 514.000 (319.000,648.000) | 441.000 (266.000,640.000) | 0.387 |
| CD8+ T cell coutns ( cells/μL) | 315.000 (239.000,419.000) | 241.000 (209.000,472.000) | 0.270 | 315.000 (252.000,419.000) | 241.000 (185.000,472.000) | 0.225 |
| NK cell counts ( cells/μL) | 276.000 (187.000,441.000) | 209.000 (177.000,300.000) | 0.386 | 276.000 (187.000,441.000) | 229.000 (177.000,439.000) | 0.736 |
| TBNK cell counts ( cells/μL) | 1364.000 (1015.000,1774.000) | 1056.000 (979.000,1579.000) | 0.319 | 1412.000 (1015.000,1774.000) | 1105.000 (979.000,1579.000) | 0.221 |
| CD3+ T cells (%) | 63.809±11.777 | 64.766±8.676 | 0.814 | 64.904±11.708 | 61.599±9.504 | 0.359 |
| B cells (%) | 10.740 (6.970,14.010) | 14.220 (7.910,16.470) | 0.325 | 10.740 (7.400,14.460) | 13.460 (6.190,15.530) | 0.650 |
| CD4+ T cells (%) | 35.094±10.561 | 37.988±5.300 | 0.244 | 36.070±9.584 | 34.581±10.351 | 0.637 |
| CD8+ T cells (%) | 23.370 (19.320,31.680) | 24.370 (17.440,28.370) | 0.849 | 23.370 (19.320,31.680) | 24.370 (17.440,28.570) | 0.800 |
| NK cells (%) | 24.328±10.960 | 22.496±10.329 | 0.641 | 23.023±10.455 | 26.467±11.507 | 0.322 |
| TBNK cells (%) | 99.480 (99.290,99.640) | 99.590 (99.410,99.760) | 0.302 | 99.480 (99.290,99.630) | 99.590 (99.360,99.800) | 0.259 |
| Th/Ts | 1.390 (0.930,2.240) | 1.550 (1.360,2.190) | 0.455 | 1.400 (1.020,2.240) | 1.500 (0.910,2.190) | 0.908 |
| CD4+ CD28+ T cells (%) | 93.330 (88.930,97.910) | 86.320 (84.130,92.210) | 0.138 | 94.300 (88.930,97.910) | 87.140 (84.130,93.330) | 0.177 |
| CD8+ CD28+ T cells (%) | 51.957±18.615 | 51.861±13.968 | 0.988 | 52.984±16.888 | 49.174±19.746 | 0.505 |
| HLADR+ CD3+ T cells (%) | 17.320 (13.170,24.340) | 16.840 (16.210,22.840) | 0.484 | 16.970 (13.170,24.340) | 18.090 (16.210,22.840) | 0.520 |
| HLADR+ CD8+ T cells (%) | 47.536±16.396 | 46.257±11.769 | 0.821 | 46.334±15.758 | 49.801±14.905 | 0.489 |
| Treg cells (%) | 2.930±1.085 | 3.127±0.770 | 0.598 | 3.046±1.059 | 2.765±0.935 | 0.397 |
| CD45RA+ Treg cells (%) | 0.430 (0.220,0.630) | 0.360 (0.310,0.620) | 0.635 | 0.440 (0.240,0.630) | 0.350 (0.190,0.620) | 0.866 |
| CD45RA- Treg cells (%)1 | 2.439±0.855 | 2.631±0.762 | 0.527 | 2.546±0.825 | 2.294±0.855 | 0.350 |
| IFN γ+ NK cells (%) | 78.923±10.027 | 78.811±10.347 | 0.977 | 78.067±10.178 | 81.019±9.544 | 0.383 |
| IFN γ+ CD8+ T cells (%) | 66.924±16.009 | 61.209±8.476 | 0.316 | 65.278±15.477 | 67.147±13.666 | 0.711 |
| IFN γ+ CD4+ T cells (%) | 26.166±10.043 | 24.779±5.115 | 0.696 | 25.183±7.629 | 27.702±12.408 | 0.420 |
| NKT cells (%) | 4.930 (3.190,7.400) | 3.560 (2.400,6.060) | 0.434 | 4.240 (3.190,7.400) | 4.530 (2.400,6.060) | 0.658 |
| NKT cell counts ( cells/μL) | 58.000 (40.000,89.000) | 58.000 (27.000,61.000) | 0.275 | 63.000 (40.000,89.000) | 58.000 (27.000,61.000) | 0.282 |
| HLADR+ CD4+ T cells (%) | 18.360 (14.610,23.680) | 21.090 (17.050,25.370) | 0.434 | 18.360 (14.610,23.200) | 21.090 (16.420,31.950) | 0.282 |
| Naïve B cells (%) | 76.010 (68.650,83.540) | 67.490 (62.040,77.890) | 0.063 | 75.750 (68.650,82.600) | 68.510 (62.830,81.780) | 0.590 |
| Memory B cells (%) | 12.230 (8.040,17.740) | 13.290 (11.760,21.080) | 0.325 | 12.630 (9.190,17.740) | 13.050 (9.880,19.580) | 0.958 |
| Unswitched B cells (%) | 5.020 (2.880,7.030) | 7.350 (6.130,16.160) | 0.012 | 5.550 (2.970,7.240) | 6.130 (3.830,14.250) | 0.286 |
| Plasma blast cells (%) | 1.660 (0.770,4.460) | 0.960 (0.460,1.670) | 0.196 | 1.920 (0.770,4.460) | 0.970 (0.460,1.670) | 0.121 |
| Naïve CD4+ T cells (%) | 28.468±12.523 | 28.117±9.661 | 0.936 | 28.004±10.929 | 29.443±14.453 | 0.710 |
| CM CD4+ T cells (%) | 31.930 (29.300,41.810) | 36.050 (28.630,42.830) | 0.896 | 33.550 (29.760,42.100) | 30.200 (27.690,37.970) | 0.306 |
| EM CD4+ T cells (%) | 33.650±10.226 | 33.725±7.323 | 0.983 | 33.395±9.355 | 34.377±10.610 | 0.753 |
| EMRA CD4+ T cells (%) | 1.190 (0.560,2.230) | 1.240 (0.920,2.100) | 0.577 | 1.190 (0.460,2.230) | 1.240 (0.920,3.270) | 0.296 |
| Naïve CD8+ T cells (%) | 12.960 (7.610,20.160) | 11.540 (7.780,16.760) | 1.000 | 13.300 (7.630,20.190) | 11.170 (7.610,16.760) | 0.375 |
| CM CD8+ T cells (%) | 2.320 (1.200,5.710) | 1.660 (0.670,2.780) | 0.213 | 2.330 (1.180,6.340) | 1.770 (1.380,2.580) | 0.277 |
| EM CD8+ T cells (%) | 43.592±14.838 | 48.225±11.392 | 0.370 | 42.975±14.648 | 48.531±12.664 | 0.225 |
| EMRA CD8+T cells (%) | 36.026±16.040 | 36.188±14.835 | 0.977 | 35.557±15.846 | 37.381±15.641 | 0.720 |
| Data are presented as number (%), X±SD, or median (25th - 75th percentile); NSCLC, non-small cell lung cancer; SCLC, small cell lung cancer; CEA, carcino-embryonic antigen; NSE, neuro-specific enolase; Cyfra21-1, cytokeratin 19; SCC, squamous cell carcinoma antigen; PD-1, programmed cell death-1; PD-L1, programmed death-ligand 1. | | | | | | |
