# Supplementary material for: Immunological profiling for short-term predictive analysis in PD-1/PD-L1 therapy for lung cancer
Source: BMC Cancer. 2024 Jul 18;24:851. doi: 10.1186/s12885-024-12628-5 (PMC11256628; doi:10.1186/s12885-024-12628-5)
Supplement: Supplementary file 2 — Supplementary Material 2 [file 12885_2024_12628_MOESM2_ESM.docx]

| Supplementary Table 2: The results of NSCLC and SCLC and different treatment after two cycle treatment. | | | | | | |
| --- | --- | --- | --- | --- | --- | --- |
| Parameters | NSCLC (n=43) | SCLC (n=12) | *p* value | PD-1 (n=42) | PD-L1 (n=13) | *p* value |
| Age (years) | 60.000 (56.000,68.000) | 59.000 (54.000,66.000) | 0.552 | 61.000 (56.000,68.000) | 60.000 (56.000,66.000) | 0.743 |
| Gender (n, %) |  |  |  |  |  |  |
| Female | 7(16.279) | 1(8.333) | 0.49 | 6(14.286) | 2(15.385) | 0.922 |
| Male | 36(83.721) | 11(91.667) |  | 36(85.714) | 11(84.615) |  |
| CEA (ng/mL) | 19.231±53.183 | 24.215±59.776 | 0.792 | 19.556±53.772 | 22.662±57.457 | 0.865 |
| NSE (ug/L) | 16.810±7.204 | 28.423±34.066 | 0.048 | 16.781±7.220 | 27.556±32.794 | 0.059 |
| Cyfra21-1 (ug/L) | 4.218±4.526 | 9.141±20.506 | 0.172 | 4.207±4.568 | 8.767±19.683 | 0.192 |
| SCC (ng/mL) | 1.056±0.349 | 0.927±0.349 | 0.290 | 1.045±0.347 | 0.975±0.370 | 0.552 |
| CD3+ T cell counts ( cells/μL) | 1110.170±443.363 | 1190.667±450.816 | 0.589 | 1101.840±444.118 | 1211.385±442.801 | 0.449 |
| B cell counts ( cells/μL) | 128.000 (82.000,168.000) | 176.000 (132.000,231.000) | 0.095 | 126.000 (82.000,168.000) | 175.000 (132.000,195.000) | 0.130 |
| CD4+ T cell+ counts ( cells/μL) | 551.000 (418.000,729.000) | 690.000 (468.000,841.000) | 0.318 | 548.000 (453.000,729.000) | 637.000 (431.000,841.000) | 0.383 |
| CD8+ T cell coutns ( cells/μL) | 407.000 (260.000,600.000) | 398.000 (337.000,515.000) | 0.839 | 399.000 (260.000,566.000) | 398.000 (337.000,561.000) | 0.858 |
| NK cell counts ( cells/μL) | 383.000 (222.000,524.000) | 278.000 (165.000,460.000) | 0.534 | 334.000 (222.000,470.000) | 450.000 (165.000,516.000) | 0.789 |
| TBNK cell counts ( cells/μL) | 1713.000 (1130.000,2064.000) | 1647.000 (1440.000,2005.000) | 0.831 | 1623.000 (1130.000,2047.000) | 1647.000 (1506.000,2082.000) | 0.470 |
| CD3+ T cells (%) | 67.118±10.449 | 68.716±10.201 | 0.646 | 67.537±10.329 | 67.242±10.691 | 0.930 |
| B cells (%) | 7.870 (5.470,10.550) | 11.480 (6.580,12.830) | 0.199 | 7.870 (5.470,11.100) | 10.240 (6.580,11.480) | 0.501 |
| CD4+ T cells (%) | 36.962±9.598 | 40.528±6.648 | 0.241 | 37.440±9.339 | 38.711±8.466 | 0.669 |
| CD8+ T cells (%) | 27.643±9.380 | 25.684±7.148 | 0.513 | 27.669±9.490 | 25.751±6.869 | 0.510 |
| NK cells (%) | 22.966±10.392 | 20.176±11.425 | 0.433 | 22.271±10.206 | 22.637±12.111 | 0.916 |
| TBNK cells (%) | 99.450 (99.230,99.640) | 99.410 (99.240,99.480) | 0.508 | 99.440 (99.210,99.640) | 99.410 (99.250,99.590) | 0.929 |
| Th/Ts | 1.410 (0.930,2.080) | 1.540 (1.260,1.730) | 0.451 | 1.410 (0.940,2.080) | 1.490 (1.190,1.730) | 0.766 |
| CD4+ CD28+ T cells (%) | 91.430 (86.370,97.640) | 90.290 (83.250,93.520) | 0.589 | 92.090 (87.050,97.720) | 89.770 (83.250,91.150) | 0.175 |
| CD8+ CD28+ T cells (%) | 49.246±19.713 | 53.401±19.237 | 0.527 | 50.731±19.993 | 48.284±18.533 | 0.702 |
| HLADR+ CD3+ T cells (%) | 25.310 (15.750,29.620) | 19.470 (18.380,25.480) | 0.548 | 25.310 (15.400,29.620) | 19.470 (18.380,25.480) | 0.642 |
| HLADR+ CD8+ T cells (%) | 53.501±16.230 | 48.116±15.874 | 0.321 | 52.735±16.940 | 51.005±13.977 | 0.744 |
| Treg cells (%) | 2.750 (2.030,3.770) | 3.020 (2.550,3.870) | 0.706 | 2.750 (2.180,3.770) | 2.650 (1.820,3.870) | 0.812 |
| CD45RA+ Treg cells (%) | 0.440 (0.260,0.620) | 0.690 (0.570,0.890) | 0.110 | 0.450 (0.260,0.620) | 0.660 (0.280,0.890) | 0.280 |
| CD45RA- Treg cells (%)1 | 2.430 (1.650,3.250) | 2.110 (1.890,2.940) | 0.846 | 2.430 (1.820,3.250) | 2.040 (1.550,2.940) | 0.446 |
| IFN γ+ NK cells (%) | 77.576±15.791 | 73.639±13.885 | 0.452 | 77.490±15.932 | 74.188±13.682 | 0.517 |
| IFN γ+ CD8+ T cells (%) | 67.911±17.752 | 59.260±16.742 | 0.15 | 65.981±19.427 | 65.418±12.570 | 0.924 |
| IFN γ+ CD4+ T cells (%) | 24.520±7.659 | 23.121±5.486 | 0.568 | 24.222±7.837 | 24.077±5.079 | 0.952 |
| NKT cells (%) | 4.780 (3.060,9.040) | 5.000 (2.160,5.710) | 0.582 | 4.170 (2.920,8.260) | 5.000 (4.390,7.190) | 0.677 |
| NKT cell counts ( cells/μL) | 73.000 (48.000,125.000) | 58.000 (48.000,101.000) | 0.514 | 71.000 (44.000,115.000) | 79.000 (55.000,151.000) | 0.566 |
| HLADR+ CD4+ T cells (%) | 22.150 (15.200,27.100) | 22.530 (16.170,24.990) | 0.976 | 21.350 (15.200,27.100) | 22.530 (18.060,24.990) | 0.789 |
| Naïve B cells (%) | 73.980 (61.720,83.090) | 67.450 (59.820,67.920) | 0.089 | 73.250 (61.720,81.780) | 67.450 (59.820,75.540) | 0.231 |
| Memory B cells (%) | 13.470 (8.860,23.750) | 21.320 (13.480,24.910) | 0.276 | 12.880 (8.860,23.750) | 20.660 (13.480,24.910) | 0.289 |
| Unswitched B cells (%) | 5.740 (3.440,8.780) | 10.000 (4.640,15.700) | 0.119 | 5.840 (3.700,10.040) | 8.160 (3.020,10.130) | 0.820 |
| Plasma blast cells (%) | 2.860 (1.390,6.080) | 3.430 (2.350,5.150) | 0.568 | 2.800 (1.450,6.080) | 3.430 (2.050,5.190) | 0.593 |
| Naïve CD4+ T cells (%) | 28.480 (19.200,35.010) | 32.270 (20.990,38.000) | 0.676 | 28.480 (19.200,35.010) | 29.800 (20.990,38.000) | 0.789 |
| CM CD4+ T cells (%) | 35.301±9.479 | 35.208±9.482 | 0.977 | 35.320±9.578 | 35.152±9.154 | 0.956 |
| EM CD4+ T cells (%) | 32.190 (27.520,39.350) | 32.930 (21.980,38.280) | 0.534 | 32.190 (26.590,39.350) | 30.120 (22.860,38.280) | 0.721 |
| EMRA CD4+ T cells (%) | 1.120 (0.590,2.450) | 1.390 (1.120,2.450) | 0.333 | 1.120 (0.590,2.310) | 1.390 (0.780,3.070) | 0.259 |
| Naïve CD8+ T cells (%) | 9.920 (5.630,13.170) | 10.390 (8.370,19.490) | 0.427 | 10.340 (6.100,17.450) | 9.890 (6.200,11.670) | 0.812 |
| CM CD8+ T cells (%) | 2.610 (1.090,5.210) | 3.220 (1.530,3.670) | 1.000 | 2.700 (1.090,5.210) | 1.840 (1.070,3.490) | 0.367 |
| EM CD8+ T cells (%) | 46.968±16.307 | 39.632±14.121 | 0.170 | 45.546±16.663 | 44.791±14.320 | 0.885 |
| EMRA CD8+T cells (%) | 31.900 (25.430,46.600) | 40.950 (28.930,44.250) | 0.632 | 31.480 (25.430,46.370) | 40.950 (28.930,48.990) | 0.422 |
| Data are presented as number (%), X±SD, or median (25th - 75th percentile); NSCLC, non-small cell lung cancer; SCLC, small cell lung cancer; CEA, carcino-embryonic antigen; NSE, neuro-specific enolase; Cyfra21-1, cytokeratin 19; SCC, squamous cell carcinoma antigen; PD-1, programmed cell death-1; PD-L1, programmed death-ligand 1. | | | | | | |
