# Supplementary material for: Immunological profiling for short-term predictive analysis in PD-1/PD-L1 therapy for lung cancer
Source: BMC Cancer. 2024 Jul 18;24:851. doi: 10.1186/s12885-024-12628-5 (PMC11256628; doi:10.1186/s12885-024-12628-5)
Supplement: Supplementary file 3 — Supplementary Material 3 [file 12885_2024_12628_MOESM3_ESM.docx]

| Supplementary Table 3: The results of NSCLC and SCLC and different treatment after four cycle treatment. | | | | | | |
| --- | --- | --- | --- | --- | --- | --- |
| Parameters | NSCLC (n=30) | SCLC (n=7) | *p* value | PD-1 (n=27) | PD-L1 (n=10) | *p* value |
| Age (years) | 61.000 (57.000,67.000) | 57.000 (54.000,63.000) | 0.371 | 61.000 (56.000,67.000) | 60.000 (54.000,69.000) | 0.797 |
| Gender (n, %) |  |  |  |  |  |  |
| Female | 8(26.667) | 1(14.286) | 0.492 | 6(22.222) | 3(30.000) | 0.624 |
| Male | 22(73.333) | 6(85.714) |  | 21(77.778) | 7(70.000) |  |
| CEA (ng/mL) | 15.456±40.694 | 3.029±2.355 | 0.438 | 16.389±42.024 | 2.991±2.118 | 0.359 |
| NSE (ug/L) | 15.408±5.562 | 14.264±5.305 | 0.635 | 15.388±5.788 | 14.579±4.625 | 0.714 |
| Cyfra21-1 (ug/L) | 3.280±2.194 | 2.620±1.218 | 0.461 | 3.413±2.248 | 2.366±0.975 | 0.196 |
| SCC (ng/mL) | 1.000±0.503 | 0.814±0.356 | 0.376 | 0.959±0.493 | 0.978±0.454 | 0.924 |
| CD3+ T cell counts ( cells/μL) | 1115.767±388.772 | 1060.286±518.513 | 0.759 | 1150.926±374.593 | 982.000±493.651 | 0.287 |
| B cell counts ( cells/μL) | 99.000 (61.000,154.000) | 125.000 (86.000,134.000) | 0.969 | 99.000 (61.000,161.000) | 120.000 (76.000,134.000) | 0.918 |
| CD4+ T cell+ counts ( cells/μL) | 545.000 (372.000,657.000) | 541.000 (540.000,623.000) | 0.801 | 585.000 (438.000,674.000) | 427.000 (212.000,623.000) | 0.281 |
| CD8+ T cell coutns ( cells/μL) | 469.600±185.699 | 387.714±238.628 | 0.342 | 472.074±189.736 | 405.600±216.089 | 0.382 |
| NK cell counts ( cells/μL) | 319.000 (205.000,437.000) | 345.000 (137.000,551.000) | 0.969 | 319.000 (164.000,437.000) | 371.000 (137.000,615.000) | 0.412 |
| TBNK cell counts ( cells/μL) | 1571.300±503.122 | 1592.000±805.556 | 0.953 | 1589.222±493.030 | 1537.400±745.392 | 0.813 |
| CD3+ T cells (%) | 70.397±10.126 | 70.691±14.913 | 0.952 | 72.111±8.304 | 65.974±15.800 | 0.290 |
| B cells (%) | 6.800 (4.160,9.990) | 5.110 (4.660,7.170) | 0.614 | 6.440 (3.920,11.170) | 6.950 (4.660,9.940) | 0.973 |
| CD4+ T cells (%) | 35.462±9.981 | 42.506±9.654 | 0.109 | 37.570±8.798 | 34.702±13.315 | 0.466 |
| CD8+ T cells (%) | 30.363±9.377 | 24.773±6.653 | 0.156 | 30.193±9.686 | 26.907±7.165 | 0.348 |
| NK cells (%) | 21.331±9.725 | 21.956±14.865 | 0.895 | 19.638±8.308 | 26.338±14.817 | 0.224 |
| TBNK cells (%) | 99.560 (99.400,99.770) | 99.320 (99.280,99.410) | 0.038 | 99.530 (99.360,99.770) | 99.440 (99.280,99.680) | 0.383 |
| Th/Ts | 1.160 (0.790,1.630) | 1.760 (1.630,1.780) | 0.071 | 1.330 (0.790,1.870) | 1.340 (1.060,1.760) | 0.973 |
| CD4+ CD28+ T cells (%) | 87.490 (83.790,96.040) | 93.410 (89.730,94.610) | 0.548 | 87.490 (82.980,96.770) | 93.410 (89.730,96.040) | 0.321 |
| CD8+ CD28+ T cells (%) | 40.003±17.416 | 53.209±16.453 | 0.085 | 41.394±16.810 | 45.492±20.572 | 0.551 |
| HLADR+ CD3+ T cells (%) | 27.702±8.189 | 21.371±5.971 | 0.069 | 27.630±7.517 | 23.466±9.148 | 0.180 |
| HLADR+ CD8+ T cells (%) | 57.446±12.563 | 50.221±15.845 | 0.215 | 56.654±12.079 | 54.528±16.782 | 0.682 |
| Treg cells (%) | 2.430 (2.110,3.150) | 2.560 (2.510,2.850) | 0.628 | 2.510 (2.110,3.360) | 2.420 (2.320,3.010) | 0.932 |
| CD45RA+ Treg cells (%) | 0.415±0.235 | 0.400±0.293 | 0.889 | 0.437±0.235 | 0.346±0.268 | 0.336 |
| CD45RA- Treg cells (%)1 | 2.130 (1.680,2.600) | 2.320 (2.230,2.540) | 0.427 | 2.160 (1.680,2.600) | 2.220 (2.010,2.570) | 0.745 |
| IFN γ+ NK cells (%) | 74.760 (62.430,85.650) | 63.350 (62.500,70.420) | 0.601 | 74.760 (60.270,85.650) | 70.420 (63.350,91.980) | 0.573 |
| IFN γ+ CD8+ T cells (%) | 71.512±14.623 | 67.046±12.233 | 0.471 | 71.127±13.541 | 69.424±16.137 | 0.756 |
| IFN γ+ CD4+ T cells (%) | 26.420 (22.900,33.100) | 29.790 (28.940,32.460) | 0.304 | 26.420 (20.870,33.100) | 28.940 (26.640,34.960) | 0.252 |
| NKT cells (%) | 5.540 (5.050,10.260) | 4.750 (4.720,7.020) | 0.313 | 5.540 (5.050,9.850) | 4.750 (4.720,11.030) | 0.694 |
| NKT cell counts ( cells/μL) | 90.000 (67.000,134.000) | 52.000 (44.000,127.000) | 0.245 | 90.000 (67.000,134.000) | 62.000 (38.000,149.000) | 0.431 |
| HLADR+ CD4+ T cells (%) | 25.450±10.036 | 21.857±8.190 | 0.397 | 24.081±8.211 | 26.632±13.025 | 0.496 |
| Naïve B cells (%) | 69.065±17.454 | 61.743±10.458 | 0.307 | 68.040±16.738 | 66.707±16.222 | 0.834 |
| Memory B cells (%) | 12.070 (7.980,23.490) | 20.920 (18.550,21.980) | 0.092 | 15.740 (7.980,23.490) | 17.200 (11.100,30.590) | 0.383 |
| Unswitched B cells (%) | 5.440 (3.380,9.460) | 7.650 (6.040,9.760) | 0.208 | 5.630 (4.400,9.460) | 6.040 (3.230,9.760) | 0.878 |
| Plasma blast cells (%) | 1.940 (1.330,6.480) | 3.470 (1.420,6.490) | 0.771 | 1.940 (1.310,6.480) | 2.230 (1.420,7.750) | 0.644 |
| Naïve CD4+ T cells (%) | 23.950 (17.610,35.230) | 24.730 (14.840,31.360) | 0.892 | 24.110 (18.690,35.230) | 14.840 (12.550,42.010) | 0.347 |
| CM CD4+ T cells (%) | 34.875±8.088 | 36.381±8.839 | 0.674 | 34.919±8.478 | 35.811±7.588 | 0.778 |
| EM CD4+ T cells (%) | 35.461±11.524 | 33.417±14.227 | 0.697 | 34.760±11.391 | 35.922±13.827 | 0.802 |
| EMRA CD4+ T cells (%) | 1.490 (0.690,3.210) | 1.070 (0.900,2.110) | 0.628 | 1.190 (0.680,3.190) | 2.110 (0.900,6.810) | 0.383 |
| Naïve CD8+ T cells (%) | 8.130 (3.510,14.980) | 11.180 (11.080,12.190) | 0.146 | 8.450 (3.510,14.980) | 11.080 (5.100,21.030) | 0.483 |
| CM CD8+ T cells (%) | 1.820 (0.720,3.270) | 2.200 (0.950,2.350) | 0.756 | 2.030 (0.860,3.090) | 0.950 (0.450,3.030) | 0.798 |
| EM CD8+ T cells (%) | 39.829±17.444 | 38.920±19.885 | 0.907 | 40.587±18.357 | 37.147±16.482 | 0.616 |
| EMRA CD8+T cells (%) | 46.087±17.550 | 41.730±20.267 | 0.580 | 45.657±17.638 | 44.199±19.514 | 0.834 |
| Data are presented as number (%), X±SD, or median (25th - 75th percentile); NSCLC, non-small cell lung cancer; SCLC, small cell lung cancer; CEA, carcino-embryonic antigen; NSE, neuro-specific enolase; Cyfra21-1, cytokeratin 19; SCC, squamous cell carcinoma antigen; PD-1, programmed cell death-1; PD-L1, programmed death-ligand 1. | | | | | | |
