# Supplementary material for: Immunological profiling for short-term predictive analysis in PD-1/PD-L1 therapy for lung cancer
Source: BMC Cancer. 2024 Jul 18;24:851. doi: 10.1186/s12885-024-12628-5 (PMC11256628; doi:10.1186/s12885-024-12628-5)
Supplement: Supplementary file 4 — Supplementary Material 4 [file 12885_2024_12628_MOESM4_ESM.docx]

| Supplementary Table 4: Comparison of baseline characteristics and counts of lymphocyte subpopulations among complete remission (CR), non-complete remission (NCR) and healthy control groups. | | | | | | | |
| --- | --- | --- | --- | --- | --- | --- | --- |
| Parameters | HC (n=20) | NCR (n=30) | CR (n=15) | P | P^a^ | P^b^ | P^c^ |
| Gender (n, %) |  |  |  |  |  |  |  |
| Female | 4 (20.000) | 3 (10.000) | 2 (13.333) | 0.603 | 0.956 | 0.615 | 0.856 |
| Male | 16 (80.000) | 27 (90.000) | 13 (86.667) |  |  |  |  |
| Age (years) | 58.000 (54.000,59.000) | 61.000 (56.000,68.000) | 60.000 (56.000,63.000) | 0.113 | 0.320 | 0.166 | 0.977 |
| Indicator |  |  |  |  |  |  |  |
| CEA (ng/mL) | 1.950 (1.730,2.280) | 4.610 (2.730,39.260) | 2.990 (1.930,3.470) | 0.001 | 0.413 | 0.256 | 0.985 |
| NSE (ug/L) | 12.770 (11.260,14.260) | 21.080 (12.710,29.210) | 17.160 (14.160,21.750) | 0.002 | 0.507 | 0.078 | 0.688 |
| Cyfra21-1 (ug/L) | 2.500 (1.930,3.330) | 4.170 (2.650,5.830) | 4.890 (2.740,8.840) | 0.004 | 1.000 | 0.123 | 0.240 |
| SCC (ng/mL) | 0.700 (0.600,0.800) | 1.100 (0.900,1.400) | 1.000 (0.700,1.600) | <0.001 | 0.438 | 0.088 | 0.778 |
| CD3+ T cell counts ( cells/μL) | 1402.000 (1180.000,1587.560) | 790.000 (655.000,1193.000) | 731.000 (548.000,1011.000) | <0.001 | 0.276 | 0.007 | <0.001 |
| B cell counts ( cells/μL) | 224.000 (176.950,273.710) | 174.000 (68.000,251.000) | 111.000 (101.000,196.000) | 0.027 | 0.682 | 0.369 | 0.14 |
| CD4+ T cell counts ( cells/μL) | 761.000 (607.000,918.270) | 504.000 (334.000,695.000) | 491.000 (311.000,598.000) | 0.003 | 0.675 | 0.047 | 0.016 |
| CD8+ T cell coutns ( cells/μL) | 571.000 (352.000,616.630) | 299.000 (217.000,474.000) | 299.000 (239.000,361.000) | 0.014 | 0.583 | 0.076 | 0.018 |
| NK cell counts ( cells/μL) | 422.000 (380.000,696.920) | 243.000 (190.000,329.000) | 359.000 (186.000,476.000) | 0.018 | 0.984 | 0.066 | 0.101 |
| TBNK cell counts ( cells/μL) | 2126.090 (1826.000,2503.000) | 1412.000 (989.000,1774.000) | 1316.000 (1023.000,1481.000) | <0.001 | 0.660 | 0.003 | 0.001 |
| CD3+ T cells (%) | 63.360 (10.889) | 63.459 (10.637) | 63.637 (12.134) | 0.997 | 0.999 | 1.000 | 0.997 |
| B cells (%) | 10.690 (8.770,12.210) | 10.620 (7.910,15.530) | 11.040 (7.420,13.960) | 0.95 | 0.813 | 0.799 | 1.000 |
| CD4+ T cells (%) | 34.863 (5.974) | 36.394 (9.108) | 35.165 (10.074) | 0.808 | 0.905 | 0.830 | 0.995 |
| CD8+ T cells (%) | 23.881 (7.701) | 24.880 (9.536) | 25.013 (8.804) | 0.911 | 0.999 | 0.929 | 0.934 |
| NK cells (%) | 24.426 (10.099) | 23.950 (10.547) | 24.972 (11.449) | 0.956 | 0.956 | 0.988 | 0.989 |
| Th/Ts | 1.440 (1.250,1.732) | 1.430 (1.030,2.380) | 1.660 (1.020,2.190) | 0.985 | 0.917 | 0.974 | 0.982 |
| CD4+ CD28+ T cells (%) | 95.200 (91.390,97.900) | 90.510 (85.970,96.600) | 97.060 (85.660,98.210) | 0.195 | 0.781 | 0.216 | 0.700 |
| CD8+ CD28+ T cells (%) | 50.670 (16.873) | 51.833 (16.956) | 53.291 (17.634) | 0.908 | 0.965 | 0.973 | 0.907 |
| HLADR+ CD3+ T cells (%) | 26.100 (18.250,30.100) | 19.060 (13.630,24.420) | 16.110 (13.170,16.970) | 0.024 | 0.492 | 0.210 | 0.038 |
| HLADR+ CD8+ T cells (%) | 40.200 (35.400,47.370) | 43.850 (35.750,56.060) | 45.590 (36.960,51.260) | 0.367 | 0.686 | 0.296 | 0.872 |
| Treg cells (%) | 1.870 (1.410,2.500) | 2.860 (2.540,3.900) | 2.610 (1.820,3.090) | <0.001 | 0.096 | <0.001 | 0.325 |
| CD45RA+ Treg cells (%) | 0.240 (0.180,0.460) | 0.350 (0.260,0.590) | 0.440 (0.240,0.620) | 0.159 | 0.799 | 0.236 | 0.708 |
| CD45RA- Treg cells (%) | 1.709 (0.660) | 2.763 (0.857) | 2.071 (0.625) | <0.001 | 0.052 | <0.001 | 0.488 |
| IFN γ+ NK cells (%) | 84.700 (79.300,89.000) | 79.100 (71.100,87.080) | 81.860 (80.730,85.760) | 0.238 | 0.425 | 0.221 | 0.978 |
| IFN γ+ CD8+ T cells (%) | 61.400 (58.260,70.800) | 68.090 (54.500,76.360) | 66.120 (59.950,73.610) | 0.717 | 0.944 | 0.910 | 0.999 |
| IFN γ+ CD4+ T cells (%) | 25.700 (18.500,29.000) | 23.920 (21.110,31.210) | 20.750 (18.420,34.450) | 0.626 | 0.836 | 0.827 | 0.998 |
| NKT cells (%) | 4.650 (3.850,8.510) | 3.860 (2.700,7.280) | 4.810 (3.660,6.340) | 0.566 | 0.857 | 0.914 | 0.684 |
| NKT cell counts ( cells/μL) | 97.000 (68.000,213.000) | 58.000 (34.000,92.000) | 58.000 (40.000,79.000) | 0.024 | 0.729 | 0.036 | 0.085 |
| HLADR+ CD4+ T cells (%) | 15.430 (13.630,18.800) | 21.290 (17.050,25.370) | 11.690 (9.760,17.070) | 0.004 | 0.047 | 0.211 | 0.708 |
| Naïve B cells (%) | 70.900 (65.500,75.640) | 75.750 (62.830,82.280) | 69.700 (68.510,81.610) | 0.388 | 0.832 | 0.896 | 0.986 |
| Memory B cells (%) | 6.010 (2.270,15.800) | 13.050 (9.880,21.080) | 13.290 (10.210,17.280) | 0.022 | 0.999 | 0.036 | 0.099 |
| Unswitched B cells (%) | 8.600 (4.240,9.230) | 5.550 (3.640,7.240) | 7.350 (5.720,9.900) | 0.226 | 0.072 | 0.902 | 0.212 |
| Plasma blast cells (%) | 0.510 (0.330,0.990) | 1.240 (0.750,3.290) | 1.310 (0.910,4.460) | 0.005 | 0.596 | 0.164 | 0.043 |
| Naïve CD4+ T cells (%) | 25.811 (14.933) | 25.917 (9.992) | 32.515 (11.181) | 0.193 | 0.244 | 1.000 | 0.286 |
| CM CD4+ T cells (%) | 32.800 (21.910,37.950) | 34.480 (29.300,46.480) | 30.300 (28.030,34.250) | 0.041 | 0.211 | 0.032 | 0.823 |
| EM CD4+ T cells (%) | 44.200 (31.390,52.540) | 34.630 (30.040,38.490) | 30.310 (24.920,37.660) | 0.117 | 0.919 | 0.089 | 0.083 |
| EMRA CD4+ T cells (%) | 1.780 (1.230,2.340) | 1.450 (0.620,2.370) | 0.920 (0.630,1.370) | 0.352 | 0.962 | 0.964 | 1.000 |
| Naïve CD8+ T cells (%) | 11.870 (4.460,21.100) | 11.540 (7.630,16.760) | 12.840 (7.610,17.710) | 0.594 | 0.672 | 0.997 | 0.676 |
| CM CD8+ T cells (%) | 1.510 (0.550,2.350) | 2.280 (1.380,6.340) | 2.320 (0.640,2.780) | 0.021 | 0.899 | 0.370 | 0.273 |
| EM CD8+ T cells (%) | 43.495 (17.293) | 45.552 (12.319) | 44.901 (19.078) | 0.906 | 0.992 | 0.905 | 0.967 |
| EMRA CD8+T cells (%) | 41.397 (17.252) | 36.252 (16.400) | 32.903 (15.395) | 0.322 | 0.818 | 0.569 | 0.337 |
| Data are presented as number (%), or median (25th - 75th percentile); HC, healthy control; CR, complete remission; NCR, non-complete remission; CEA, carcino-embryonic antigen; NSE, neuro-specific enolase; Cyfra21-1, cytokeratin 19; SCC, squamous cell carcinoma antigen; P, comparison among the three groups of HC, CR, and NCR; P^a^, comparison between CR and NCR groups; P^b^, comparison between HC and NCR groups; P^c^, comparison between HC and CR groups. | | | | | | | |
