# Supplementary material for: Immunological profiling for short-term predictive analysis in PD-1/PD-L1 therapy for lung cancer
Source: BMC Cancer. 2024 Jul 18;24:851. doi: 10.1186/s12885-024-12628-5 (PMC11256628; doi:10.1186/s12885-024-12628-5)
Supplement: Supplementary file 5 — Supplementary Material 5 [file 12885_2024_12628_MOESM5_ESM.docx]

| Supplementary Table 5: The results of the ROC. | | | | |
| --- | --- | --- | --- | --- |
| Predictor | AUC (95% CI) | Sensitivity | Specificity | Cut-off |
| Treg cells % | 0.688 (0.510-0.841) | 0.467 | 1.000 | 3.43% |
| HLA-DR+ CD4+ T cells % | 0.759 (0.583-0.916) | 0.900 | 0.667 | 15.36% |
| Naive CD4+ T cells % | 0.674 (0.498-0.829) | 0.933 | 0.433 | 21.97% |
| AUC, The area under the ROC curve; CI, confidence interval. | | | | |
